# Hypomagnetic Field and Its Effect on the Growth and Survival of Microorganisms

**DOI:** 10.3390/microorganisms13061362

**Published:** 2025-06-12

**Authors:** Miroslava Sincak, Kateřina Benediktová, Jana Adámková, Jana Sedlakova-Kadukova

**Affiliations:** 1 Department of Environmental Sciences, Institute of Chemistry and Environmental Sciences, University of Ss. Cyril and Methodius in Trnava, Nam. J. Herdu 2, 91701 Trnava, Slovakia; sincak1@ucm.sk; 2 Faculty of Forestry and Wood Sciences, Czech University of Life Sciences Prague, Kamycka 129, Praha 6—Suchdol, 16500 Praha, Czech Republicadamkovaj@fld.czu.cz (J.A.); 3 ALGAJAS s.r.o., Prazská 16, 04011 Kosice, Slovakia

**Keywords:** hypomagnetic field, microorganisms, growth inhibition, environmental stress, yeast, bacteria

## Abstract

As humanity embarks on interplanetary exploration and envisions future colonies beyond Earth, understanding the impact of extreme environments on life becomes paramount. Among these factors, the hypomagnetic field (HMF)—a condition where the protective geomagnetic field is absent—remains poorly understood, especially regarding its effects on (micro)organisms. To our knowledge, this is the first study to examine how short-term exposure to an HMF (24 h to 7 days) affects the growth of three different microorganisms, *Saccharomyces cerevisiae*, *Acidithiobacillus ferrooxidans*, and *Lactobacillus plantarum*, using a specialized hypomagnetic chamber and advanced spectrophotometric analysis. We demonstrate significant growth inhibition in *S. cerevisiae* (23%) and *A. ferrooxidans* (68%), with *L. plantarum* remaining unaffected. This inhibitory effect appears reversible, diminishing as organisms return to normal geomagnetic conditions. These findings reveal that the HMF acts as a temporary environmental stressor, underscoring the need for deeper exploration of its biological effects. Our work sets the stage for further research into how the space environment may shape microbial ecosystems critical to future human endeavors in space.

## 1. Introduction

The geomagnetic field generated by the Earth is not merely a geophysical phenomenon; it plays a fundamental and often underappreciated role in shaping the biological activity and evolution of life on our planet. As an ever-present abiotic environmental factor, the geomagnetic field has been a constant companion throughout evolutionary history, subtly influencing the development, adaptation, and functioning of all living organisms. Its presence is so integral that any deviations from normal geomagnetic conditions—such as reductions, fluctuations, or complete absence—can have measurable consequences on biological systems. One such condition is referred to as a hypomagnetic field (HMF), which describes an environment where the intensity of the geomagnetic field is substantially diminished or entirely absent. These hypomagnetic conditions are known to perturb normal physiological and metabolic processes across a broad spectrum of life forms, although the extent and nature of these effects can vary significantly between organisms [1].

Although numerous investigations have been conducted on the effects of enhanced or artificially modified magnetic fields—particularly those stronger than the Earth’s natural geomagnetic field—the scientific exploration of weakened or near-zero magnetic field environments remains limited in scope [2,3]. HMFs are not purely theoretical or laboratory constructs; they are characteristic of several real-world contexts. For instance, such conditions are prevalent in interplanetary space, where planetary magnetic protection is minimal or absent, and on celestial bodies within our solar system that either lack a magnetic field entirely or possess one significantly weaker than Earth’s [4]. In more terrestrial scenarios, localized hypomagnetic conditions can also emerge in urban environments, especially in buildings constructed with steel or other materials that provide substantial magnetic shielding. These environments can lead to microzones where the geomagnetic field is reduced, affecting the organisms residing or operating within them [5].

Functionally, the geomagnetic field is believed to interact with living systems at the biochemical level, particularly by modulating redox reactions and influencing the behavior of charged particles such as ions and free radicals. These interactions are crucial for various fundamental cellular processes, including but not limited to electron transport chains in mitochondria and bacteria, oxidative stress responses, and the regulation of intracellular ion concentrations [1]. When organisms are placed in hypomagnetic environments—whether through artificial simulation or by natural occurrence in shielded spaces or extraterrestrial contexts—these regulatory processes may be disrupted. Such disruptions can lead to a cascade of physiological changes, ranging from altered metabolic rates and impaired energy production to broader systemic effects.

Of particular interest are microorganisms, whose high adaptability, metabolic diversity, and short generation times make them highly suitable for studying these subtle and complex biological effects. Unlike multicellular organisms, microbes can rapidly adjust to environmental stressors and display quantifiable physiological changes that can be measured with precision. Because of this, they serve as ideal models to dissect the potential implications of geomagnetic field absence. Microorganisms are especially important in scenarios such as underground infrastructure, closed-system bioreactors, and spaceflight habitats, where magnetic shielding might influence microbial viability and function [5].

To date, the majority of HMF-related biological research has concentrated on higher organisms such as plants and animals. In these groups, documented outcomes of exposure to hypomagnetic conditions include suppressed cellular respiration [6], diminished ATP generation [7], stunted growth in vegetative tissues [8,9], and altered behavioral or neurological functions in animals, including memory and cognition [4,10]. In contrast, investigations into microbial responses under hypomagnetic conditions are surprisingly scarce. The few existing studies have typically examined specialized phenomena such as altered antibiotic susceptibility [11] or structural adaptations in magnetotactic bacteria, such as changes in magnetosome size and morphology [12]. One of the very few reports examining microbial communities under prolonged HMF exposure revealed shifts in gut microbial diversity in mice, suggesting that even host-associated microbiomes may respond to magnetic deprivation [13].

The scarcity of detailed studies on microbial responses to hypomagnetic fields is a significant gap in our understanding—especially given the critical role microorganisms play in both ecological and biotechnological systems. This gap is particularly important to address in the context of space exploration and potential colonization of other planets, where humans and their microbial symbionts will inevitably encounter environments with weak or absent magnetic fields. Beyond extraterrestrial applications, there are also pressing Earth-based implications. For example, densely built urban areas with thick concrete or metal infrastructure can unintentionally shield geomagnetic fields, creating unintended HMF zones. Within such environments, microbial populations may behave differently, which could affect processes like water treatment, fermentation, or biofouling. These shifts could in turn influence industrial yields or environmental safety [14]. Furthermore, biotechnological processes that rely on microbial fermentation or biocatalysis might unknowingly be affected by local geomagnetic anomalies. Understanding how microbes respond to such magnetic disturbances could enable the design of more robust and predictable microbial systems for industrial use.

The biological mechanisms by which organisms detect and respond to magnetic fields are still under investigation. Several hypotheses have been proposed, though none are universally accepted. Among these are models involving superparamagnetic particles within cells, behaviors of paramagnetic ions, or the quantum effects of electron spin states. All these could theoretically allow cells to sense and respond to geomagnetic cues [5]. Additional ideas suggest the involvement of redox-active metal–ligand complexes and specific biochemical pathways involving oxidation–reduction reactions [15,16,17]. Despite these theories, the precise cellular and molecular targets of hypomagnetic field exposure remain elusive, and further empirical data are required to determine which of these mechanisms are biologically relevant.

To address some of these knowledge gaps, we designed a study focusing on the short-term effects of HMF exposure on three well-characterized microbial species, each representing different phylogenetic groups and metabolic strategies. The organisms selected were *Acidithiobacillus ferrooxidans*, a Gram-negative, chemolithoautotrophic bacterium known for its iron-oxidizing capacity; *Lactobacillus plantarum*, a Gram-positive facultative anaerobe frequently used in food fermentation; and *Saccharomyces cerevisiae*, a model eukaryotic yeast with widespread industrial and laboratory relevance. The goal of the study was to assess whether exposure to a hypomagnetic environment would impair microbial growth and whether different species exhibit varying degrees of sensitivity to this stressor. Given prior findings in non-microbial systems, we hypothesized that even short-term exposure—ranging from 24 h to 7 days—would be sufficient to induce measurable disruptions in growth and metabolic activity, especially in organisms dependent on redox processes for energy metabolism.

## 2. Materials and Methods

### 2.1. Experimental Organisms

The baker’s yeast *S. cerevisiae* (CCY 21-4-13, distillery culture) was obtained from the collection of the Chemical Institute of the Slovak Academy of Sciences (SAV) in Bratislava. The bacterium *A. ferrooxidans* was obtained from the bacterial collection of the Institute of Geotechnics of SAV in Košice, and *L. plantarum* was obtained from the bacterial collection of Pavol Jozef Safarik University in Kosice.

The *S. cerevisiae* culture was maintained on Minimal Defined medium (MD) agar (61 g/1000 mL of distilled H_2_O, composition: 20 g/L malt extract, 20 g/L dextrose, 15 g/L agar) (Sigma-Aldrich, St. Louis, MO, USA). For liquid cultures, we used Yeast extract Peptone Dextrose medium (YPD) (1% (*w*/*v*) yeast extract, 2% (*w*/*v*) peptone, and 2% (*w*/*v*) glucose) and MD agar (1.34% yeast nitrogen base, 5% biotin, 2% dextrose) (all from Sigma-Aldrich, St. Louis, MO, USA) in distilled water. The bacteria were cultivated in liquid media: *A. ferrooxidans* in liquid 9K medium (solution A: 3 g (NH_4_)_2_SO_4_/L, 0.5 g K_2_HPO_4_/L, 0.5 g MgSO_4_/L, 0.1 g KCl/L, 0.01 g Ca(NO_3_)_2_/L; solution B: 44.7 g FeSO_4_·7H_2_O/L, all from Sigma-Aldrich, St. Louis, MO, USA) and *L. plantarum* in liquid MRS medium (composition: 16.35% universal peptone, 8.18% meat extract, 8.18% yeast extract, 32.71% D(+)-glucose, 3.27% dipotassium hydrogen phosphate, 3.27% triammonium citrate, 8.18% sodium acetate, 0.16% magnesium sulfate, 0.08% manganous sulfate monohydrate, 19.62% agar, all from Sigma-Aldrich, St. Louis, MO, USA).

### 2.2. Magnetic Field Induction

A pair of Helmholtz electromagnetic coils in each electromagnetic chamber, equipped with a triaxial magnetic field compensation system (Stefan Mayer Instruments GmbH & Co. KG, Dinslaken, Germany), was used to generate a region of a uniform static electromagnetic field (EMF). This system allows for the adjustment of both the intensity and direction of the EMF. The diameter of the EMF coils was 2 m × 2 m × 2 m, and experimental cultures were placed on a table at the center of each coil, where the field was homogeneous. In one coil, a near-zero EMF (0 μT) was set, while in the other, Earth’s geomagnetic field (49 μT) was simulated.

### 2.3. Experimental Design

Yeast stock culture (*S. cerevisiae*) was inoculated into YPD medium and pre-cultivated for 18 h at 28 °C under static conditions (no agitation) in the dark. The pre-cultures were used to initiate biologically independent experimental and control cultures. Specifically, three independent pre-cultures were prepared for each organism, and each was used to inoculate one biological replicate of the control and one of the hypomagnetic field (HMF) treatment group, ensuring proper replication and avoiding pseudo-replication.

All cultures were cultivated in open, static 15 mL plastic containers (containing 10 mL of medium) without agitation or additional aeration, to maintain a restricted oxygen supply. Containers were kept in complete darkness at a constant room temperature of 28 ± 1 °C throughout the experiment.

**Figure 1:**
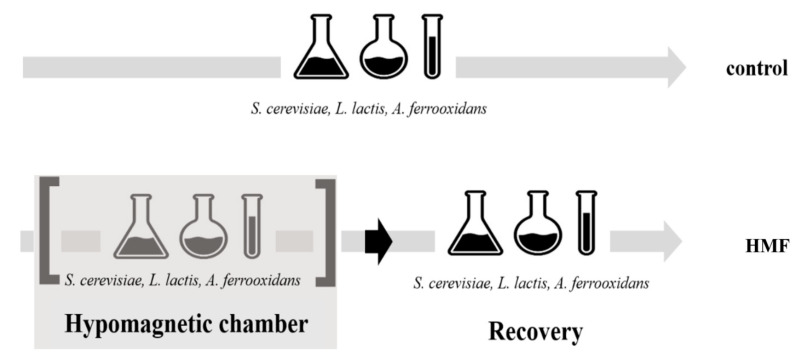
The experimental design for hypomagnetic field (HMF) exposure and recovery. *S. cerevisiae* and *L. plantarum* were exposed for 24 h, while *A. ferrooxidans* was exposed for 7 days due to its slower growth rate. Following HMF exposure, all cultures underwent a recovery period under standard laboratory conditions.

Experimental cultures were exposed to a hypomagnetic field (HMF), while control cultures were maintained at background geomagnetic field levels (see Figure 1). The exposure duration to HMF was 24 h for *S. cerevisiae* and *L. plantarum* and 7 days for *A. ferrooxidans*, due to its slower growth rate. Following HMF exposure, cultures were transferred back to background magnetic conditions and maintained under the same static cultivation parameters for an additional 24 h (*S. cerevisiae* and *L. plantarum*) or 7 days (*A. ferrooxidans*) as a recovery phase.

The initial optical density (OD_600_) at inoculation was adjusted to approximately 0.1 for *S. cerevisiae* and *L. plantarum* and to 0.05 for *A. ferrooxidans*. All experiments were performed in biological triplicates. Error bars in the results represent the standard deviation (SD) of the biological replicates. The control variants remained under laboratory conditions throughout the entire experiment (48 h for *S. cerevisiae* and *L. plantarum*, 14 days for *A. ferrooxidans*). The longer cultivation time for *A. ferrooxidans* was necessary due to its slower growth rate. Measurements of pH, redox potential, cell counting, and viability assessment were performed at regular time intervals (Figure 1).

### 2.4. Measured Parameters

pH and redox potential were measured using a pH meter (Xylem Analytics Germany GmbH, Weilheim, Germany). Before measuring pH and redox potential, the culture was filtered through a 0.22 μm cell filter (Techno Plastic Products AG, Trasadingen, Switzerland) to increase measurement stability. Growth was evaluated using a growth curve constructed by spectrophotometric measurement (WPA Biowave 3) at 430 nm (*A. ferrooxidans*) and 600 nm (*L. plantarum*) and by direct cell counting using an improved Neubauer chamber (*A. ferrooxidans*, *L. plantarum*). All measurements of pH, redox potential, growth, and survival were performed in triplicate.

The growth and viability of *S. cerevisiae* were assessed using a Bürker counting chamber and colony-forming unit (CFU) counts on MD agar plates. The percentage of budding yeast cells (cells containing at least one bud) was also evaluated. Statistical analyses were carried out using Social Science Statistics software (https://www.socscistatistics.com/, non-versioned web tool).

Statistical analysis was performed using a two-tailed unpaired Student’s *t*-test to compare the means of control and treatment groups. Prior to applying the *t*-test, data were assessed for normality using the Shapiro–Wilk test and for the homogeneity of variances using Levene’s test. Results are reported as mean ± standard deviation (SD) from biological triplicates. No formal correction for multiple comparisons was applied, as each comparison was predefined and limited to direct pairwise testing between HMF-exposed and control groups for each organism and time point.

## 3. Results

### 3.1. Organisms in Hypomagnetic Field

#### 3.1.1. *Saccharomyces cerevisiae*

**Figure 2:**
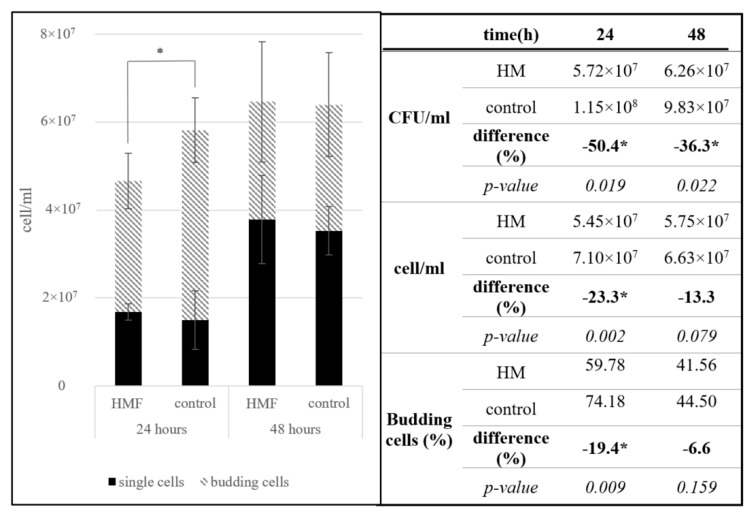
Growth response of *S. cerevisiae* under hypomagnetic field (HMF) conditions. Significant reductions in cell viability, total cell count, and budding cell percentage were observed during HMF exposure. However, after 24 h of recovery, values returned to control levels, indicating that the effect is temporary. * The difference was significant according to a double-tailed *t*-test (*p* < 0.05).

After 24 h in a hypomagnetic field, we observed a significant reduction in cell viability, budding cell count, and total cell count in *S. cerevisiae*, ranging from 19% to 56%. These changes were most pronounced immediately after withdrawal from the hypomagnetic field. During the 24 h recovery period under laboratory conditions, this difference diminished, and after 24 h in standard conditions, no significant difference was detected (Figure 2).

In the initial 24 h of exposure to a hypomagnetic field, the number of budding cells decreased significantly by 19%. The reduced budding cell count, along with lower cell viability measured by CFU/mL, indicates that the hypomagnetic field not only decelerates growth but also significantly limits cell propagation.

The graphical representation in Figure 2 clearly illustrates the transient nature of this inhibitory effect. During the hypomagnetic exposure, a sharp decline is observed across all growth indicators—total cell number, budding cell percentage, and viability—demonstrating an acute inhibitory effect of the hypomagnetic field. Notably, the fact that budding cells are disproportionately affected suggests that cell division processes are particularly sensitive to magnetic deprivation, possibly due to disruptions in cytoskeletal dynamics, membrane potential, or mitochondrial activity during the budding cycle. The recovery phase depicted in the graph shows a near-complete rebound in all parameters after 24 h under normal geomagnetic conditions. This not only confirms the reversibility of the HMF-induced effects but also implies that *S. cerevisiae* can rapidly reestablish homeostasis when environmental magnetic inputs are restored. Such resilience may be advantageous in fluctuating environments and is particularly relevant for spaceflight or closed bioreactor systems, where brief interruptions in magnetic exposure could occur.

Several studies have investigated the effects of magnetic fields stronger than Earth’s geomagnetic field on microorganisms, particularly yeast, due to their relevance in industrial applications. Various authors have reported either inhibitory effects [18,19,20] or no significant impact [21,22] of magnetic fields on yeast. Positive effects of magnetic fields have been observed only under specific conditions, such as small culture volumes and electromagnetic fields ranging from 0.5 mT to 25 mT [23,24,25,26] or with stronger magnetic fields induced by permanent magnets [27].

To date, only one study has explored the effects of hypomagnetic fields (BAC = 0.365 µT, F = 50 Hz) on yeast growth. Bajtos et al. [28] reported inhibition of *S. cerevisiae* growth in 24 out of 25 experiments. The authors suggested that the hypomagnetic field may influence yeast cells through a feedback mechanism, in which alterations in the frequency, phase, or amplitude of the electromagnetic field could modulate yeast behavior. However, the study did not provide a detailed explanation of the cellular mechanisms underlying magnetoreception under hypomagnetic conditions.

The exact mechanism by which yeast detects and responds to different magnetic field conditions remains unclear. Proposed mechanisms include effects on the plasma membrane and ion transport [29], disruptions in energy metabolism involving ATP synthesis [30], and changes in enzyme activities and gene expression modulation [20]. It is plausible that the observed effects of hypomagnetic fields result from a combination of these mechanisms. However, the relative contribution of each mechanism to the overall response remains unknown and warrants further investigation.

#### 3.1.2. *Acidithiobacillus ferrooxidans*

**Figure 3:**
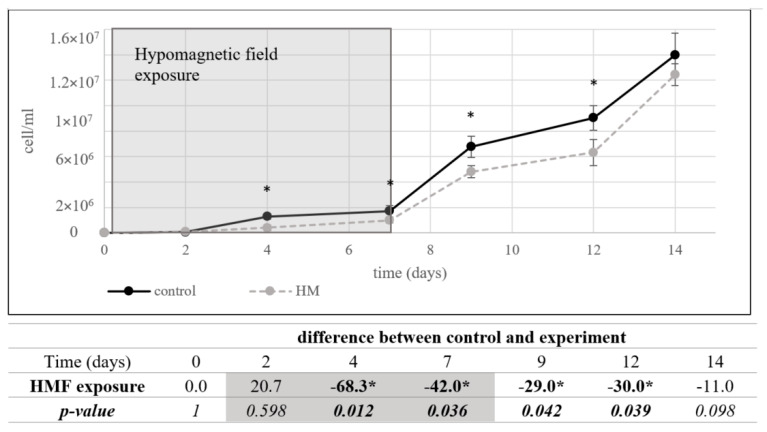
The cell counts of *A. ferrooxidans* after 7 days of hypomagnetic field (HMF) exposure followed by 7 days under laboratory conditions. Control cultures were exposed only to the geomagnetic field. After 7 days of cultivation, both control and experimental cultures were transferred to the same laboratory conditions for an additional 7 days. A significant reduction in cell count was observed in the HMF-exposed culture during the first 7 days (*: *p* < 0.05, two-tailed *t*-test), as indicated by asterisks. The table shows the percentage difference between control and experimental groups and corresponding *p*-values.

The inhibitory effect of the hypomagnetic field was more pronounced in *A. ferrooxidans* than in *S. cerevisiae*. During the seven-day hypomagnetic exposure, growth inhibition ranged from 42% to 68%, with significant effects observed as early as day four. After removal from the hypomagnetic conditions, the bacterial culture showed no significant growth difference (11%) after seven days in standard laboratory conditions, suggesting a slow recovery (Figure 3).

As in the case of *S. cerevisiae*, studies investigating the effects of stronger-than-geomagnetic fields on *A. ferrooxidans* have described positive influences on growth and bioleaching ability under moderately strong magnetic fields (3.15–9.6 mT) [31,32]. These findings suggest that magnetic fields can modulate the metabolic activity of this species. The absence of the geomagnetic field, as an environmental factor, appears to be perceived by *A. ferrooxidans*, leading to significant growth inhibition after only four days of exposure.

One possible explanation for this sensitivity is the bacterium’s dependence on iron oxidation for energy production. *A. ferrooxidans* is a chemoautotrophic organism that derives energy from metal ions through redox reactions, a process potentially influenced by external magnetic fields. The hypomagnetic field may disrupt ion transport, altering energy metabolism and affecting cellular proliferation.

Following hypomagnetic exposure, *A. ferrooxidans* exhibited slow improvement in growth parameters over an additional seven days under normal geomagnetic conditions. This suggests that while hypomagnetic exposure acts as an environmental stressor, it does not induce permanent alterations in the bacterium’s metabolic capacity.

Previous studies on magnetotactic bacteria, which form magnetosomes for navigation along Earth’s geomagnetic field, have shown that hypomagnetic field conditions (500 nT) resulted in the production of larger magnetosomes, accompanied by growth inhibition [12,33]. This suggests that bacterial responses to magnetic fields may involve metabolic adaptations linked to iron metabolism. Although *A. ferrooxidans* also has the capacity to form magnetosomes [34], their production is typically restricted to genetically modified strains and/or specific environmental conditions [35,36]. Therefore, magnetosomes are unlikely to be the primary target of hypomagnetic fields in *A. ferrooxidans* under our experimental conditions. Instead, hypomagnetic fields may have influenced other naturally occurring processes, such as ion uptake and energy metabolism [37,38].

The observed delay in *A. ferrooxidans* growth under hypomagnetic conditions may stem from impaired efficiency in its iron oxidation pathway, which is central to its energy metabolism [37]. As a chemoautotroph, *A. ferrooxidans* relies on the oxidation of ferrous to ferric iron to drive proton motive force generation and ATP synthesis [29,30]. The absence of a magnetic field could potentially interfere with electron transfer processes involved in this redox cycle, either by affecting the orientation or behavior of iron ions or by disrupting radical pair dynamics in redox-active enzymes. Moreover, slower proliferation may reflect a cellular adaptation strategy, wherein metabolic rate and division are downregulated under energetically unfavorable conditions. This temporary metabolic suppression could help the cells conserve resources and maintain essential functions while the external magnetic conditions remain suboptimal. Upon reintroduction to geomagnetic conditions, the reactivation of iron-oxidation-related pathways likely underpins the gradual recovery observed, underscoring the resilience of this bacterium despite environmental stress.

#### 3.1.3. *Lactobacillus plantarum*

**Figure 4:**
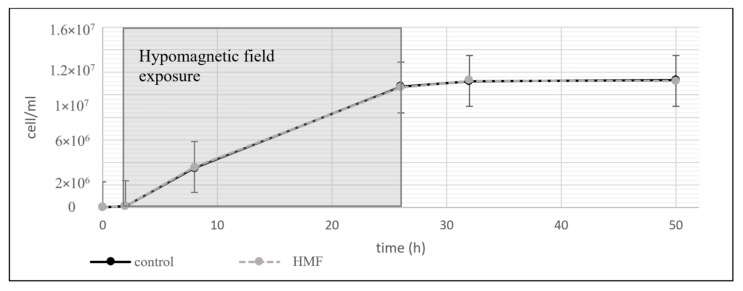
Growth curve of *L. plantarum* under hypomagnetic field (HMF) exposure. Control cultures were maintained under the geomagnetic field, while experimental cultures were exposed to HMF for the first 28 h (shaded area). Afterward, both groups were cultivated under identical laboratory conditions. No significant differences in cell counts were observed, indicating that *L. plantarum* growth is not notably affected by the absence of the geomagnetic field.

In contrast to *S. cerevisiae* and *A. ferrooxidans*, *L. plantarum* showed no discernible changes in growth or viability under hypomagnetic field exposure (Figure 4).

The apparent insensitivity of *L. plantarum* to the hypomagnetic field may be attributed to its metabolic properties. Unlike *A. ferrooxidans*, which depends on metal ions for energy production, or *S. cerevisiae*, which requires iron as a mitochondrial cofactor, *L. plantarum* does not require iron or other paramagnetic particles for essential metabolic functions [39,40]. The reduced dependence on Fe-S cofactors, which are present in both *S. cerevisiae* and *A. ferrooxidans*, further supports the hypothesis that paramagnetic ion metabolism plays a role in hypomagnetic field sensitivity.

To date, no studies have specifically examined the effects of hypomagnetic fields on Lactobacillus species. Most research on bacterial responses to magnetic fields has focused on Gram-negative bacteria, particularly magnetotactic bacteria [41]. A recent study by Ilyin et al. [42] investigated bacterial isolates from the nasopharynx of cosmonauts after space missions and observed changes in microbial populations, including reduced antibiotic resistance. Although this study did not directly examine the role of hypomagnetic fields, it raises the possibility that bacterial physiology may be affected by prolonged exposure to low magnetic field environments.

### 3.2. Comparison of Experimental Organisms

Throughout cultivation in the hypomagnetic field, no significant changes in pH or redox potential were observed in any of the tested microorganisms compared to their respective controls (Table 1).

When comparing the effects of the hypomagnetic field on the three experimental organisms, notable differences were observed. The growth of *A. ferrooxidans* was significantly inhibited, with reductions of up to 68%, while *S. cerevisiae* exhibited milder inhibition of approximately 23%. In contrast, *L. plantarum* showed no significant response to the hypomagnetic conditions. The observed redox shift in *A. ferrooxidans* under hypomagnetic field exposure (Table 1), while not statistically significant, may indicate the initiation of early stress signaling mechanisms and thus warrants further investigation.

Our study demonstrated that hypomagnetic fields can influence the growth of certain microorganisms, both eukaryotic and prokaryotic, with the effect being highly organism-dependent. This variability may result from differences in magnetic sensitivity among microorganisms, likely influenced by their distinct metabolic requirements and environmental interactions.

Interestingly, while *A. ferrooxidans*, as a chemoautotrophic bacterium, relies on metal ions for energy production through redox reactions [43], and *S. cerevisiae* depends on iron cofactors in mitochondria [44], *L. plantarum* does not require metal ions or Fe-S cofactors [39,40]. These observations suggest that the differing responses to hypomagnetic conditions may be related to paramagnetic particle metabolism, particularly metal ion uptake, emphasizing ion transport as a potentially critical mechanism for hypomagnetic reception.

Further investigation into various magnetoreception mechanisms, including the effects on ion transport [29], energy metabolism involving ATP synthesis [30], and changes in enzyme activities or gene expression modulation [20], will be essential to elucidate the exact mechanisms and the relative contributions of each to the overall response.

Our findings provide a valuable framework for understanding how specific physiological traits—such as dependence on iron–sulfur clusters or redox-based metabolism—correlate with susceptibility to hypomagnetic environments. It is worth considering that many microbial taxa not examined here may show even greater variation in response. For example, magnetotactic bacteria might exhibit drastically different behavior under near-zero magnetic conditions, possibly disrupting magnetosome synthesis or navigation. Similarly, extremophilic organisms adapted to high-radiation or high-pressure environments might possess unique magnetic field tolerances. Future studies expanding this organismal spectrum are likely to yield additional mechanistic insights.

## 4. Conclusions

This study provides evidence that hypomagnetic fields influence microbial growth in a species-dependent manner. While *S. cerevisiae* and *A. ferrooxidans* exhibited significant growth inhibition under hypomagnetic conditions, *L. plantarum* remained unaffected. These findings suggest that sensitivity to hypomagnetic fields may be associated with metabolic pathways involving paramagnetic ions, particularly iron metabolism.

Growth inhibition in *S. cerevisiae* and *A. ferrooxidans* was reversible, as both organisms demonstrated recovery after re-exposure to Earth’s geomagnetic field. This indicates that hypomagnetic fields act as temporary stressors rather than inducing permanent metabolic damage.

This study also suggests potential practical implications for microbial life support systems in space stations or long-duration missions. Maintaining minimal geomagnetic field simulation might be necessary to ensure stable microbial processes, especially those involving fermentation, waste recycling, or bioremediation. Artificial magnetic shielding or field generators could become standard components of extraterrestrial bioreactors. Additionally, microbial community engineering efforts could benefit from screening strains not only for thermal or pH tolerance but also for magnetic field resilience.

Further research is needed to explore the mechanisms underlying microbial magnetoreception and the potential applications of hypomagnetic field exposure in microbiology and biotechnology. Understanding how microorganisms respond to low-magnetic environments is particularly relevant for space exploration and extraterrestrial habitat development, where hypomagnetic conditions are prevalent.

It is also noteworthy that while *L. plantarum* showed no measurable difference in growth, this does not rule out the possibility of changes in gene expression or intracellular signaling.

Furthermore, the rapid recovery of *S. cerevisiae* and *A. ferrooxidans* post-exposure suggests a dynamic and reversible adaptation process. The temporary suppression of growth may be due to disruptions in membrane potential or enzyme function, both of which are sensitive to changes in magnetic fields. Once returned to geomagnetic conditions, the restoration of growth implies a robust homeostatic mechanism that compensates for short-term stress. Investigating this recovery phase in more detail—perhaps using real-time metabolic flux analysis—could provide clues about the cellular pathways most affected by magnetic deprivation.

Lastly, interspecies differences in magnetic field response could be harnessed in co-culture or consortia-based biotechnology. Understanding how each species behaves in isolation under HMF conditions lays the groundwork for evaluating mixed cultures, where synergistic or antagonistic interactions could either buffer or amplify magnetic effects. This knowledge could guide the design of magnetically modulated microbial communities for bioproduction, waste treatment, or life-support systems in low-field environments such as space habitats.

## Figures and Tables

**Table 1:** Mean ± standard deviation of pH and redox potential of control and hypomagnetic-field-exposed cultures after 3 biological replicates.

	pH		Redox Potential (mV)	
	Control	Hypomagnetic Field	Control	Hypomagnetic Field
*S. cerevisiae*	5.3 ± 0.1	5.3 ± 0.1	92.3 ± 1.5	100.3 ± 1.2
*A. ferrooxidans*	2.3 ± 0.05	2.2 ± 0.1	421.2 ± 3.1	408.0 ± 4.2
*L. plantarum*	3.8 ± 0.15	3.8 ± 0.1	101.9 ± 1.8	99.4 ± 2.0

## Data Availability

The original contributions presented in this study are included in the article. Further inquiries can be directed to the corresponding author.
